# Supplementary figures and images for: Inflammatory osteolysis is regulated by site-specific ISGylation of the scaffold protein NEMO
Source: eLife. 2020 Mar 23;9:e56095. doi: 10.7554/eLife.56095 (PMC7145425; doi:10.7554/eLife.56095)

**Figure 3S**

**Mem 1.1 (10%)**

**IB: Rb phos-p65 (Licor 800)**

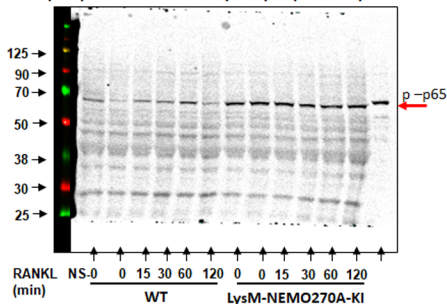

**Mem 1.2 (10%)**

**IB: Rb total-p65 (Licor 800)**

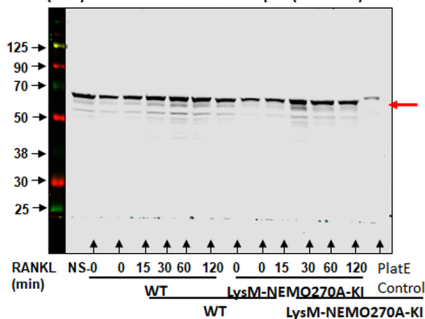

**Mem 1.4 (10%)**

**IB: ACTIN (Licor 680 only)**

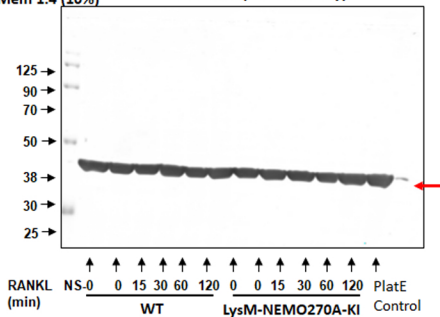

Supplement: Figure 1—source data 1. [file elife-56095-fig1-data1.pdf]

**Figure 4E**

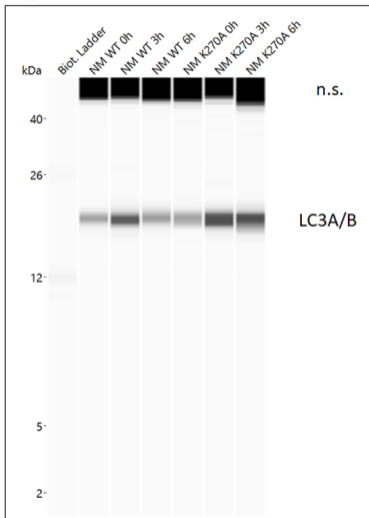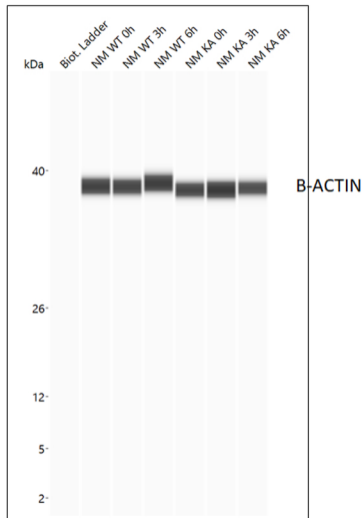

Supplement: Figure 4—source data 1. [file elife-56095-fig4-data1.pdf]

**Figure 7I**

**Broad-range separation**

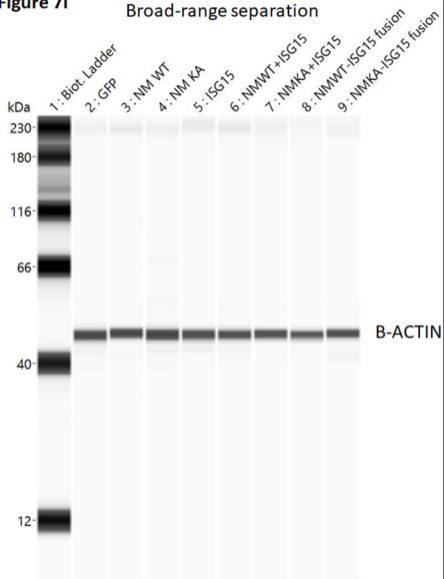

**LMW separation**

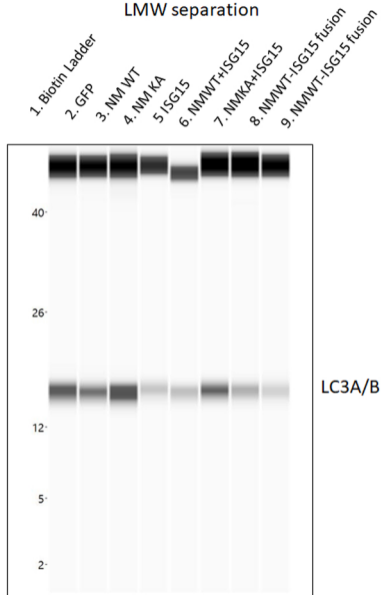

Supplement: Figure 7—source data 5. [file elife-56095-fig7-data5.pdf]
