# Supplementary material for: Inflammatory osteolysis is regulated by site-specific ISGylation of the scaffold protein NEMO
Source: eLife. 2020 Mar 23;9:e56095. doi: 10.7554/eLife.56095 (PMC7145425; doi:10.7554/eLife.56095)
Supplement: Figure 6—source data 2. [file elife-56095-fig6-data2.pdf]

Figure 6C

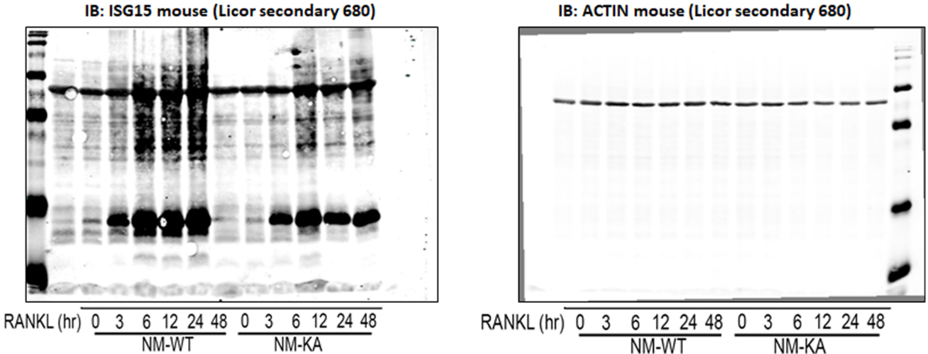
IB: ISG15 mouse (Licor secondary 680)
IB: ACTIN mouse (Licor secondary 680)
RANKL (hr) 0 3 6 12 24 48 0 3 6 12 24 48
NM-WT
NM-KA
RANKL (hr) 0 3 6 12 24 48 0 3 6 12 24 48
NM-WT
NM-KA
